# Exploring Entrainment Patterns of Human Emotion in Social Media

**DOI:** 10.1371/journal.pone.0150630

**Published:** 2016-03-08

**Authors:** Saike He, Xiaolong Zheng, Daniel Zeng, Chuan Luo, Zhu Zhang

**Affiliations:** 1 State Key Laboratory of Management and Control for Complex Systems, Institute of Automation, Chinese Academy of Sciences, Beijing, 100190, China; 2 Department of Management Information Systems, University of Arizona, Tucson, Arizona, 85721, United States of America; Instituto de Fisica Interdisciplinar y Sistemas Complejos IFISC (CSIC-UIB), SPAIN

## Abstract

Emotion entrainment, which is generally defined as the synchronous convergence of human emotions, performs many important social functions. However, what the specific mechanisms of emotion entrainment are beyond in-person interactions, and how human emotions evolve under different entrainment patterns in large-scale social communities, are still unknown. In this paper, we aim to examine the massive emotion entrainment patterns and understand the underlying mechanisms in the context of social media. As modeling emotion dynamics on a large scale is often challenging, we elaborate a pragmatic framework to characterize and quantify the entrainment phenomenon. By applying this framework on the datasets from two large-scale social media platforms, we find that the emotions of online users entrain through social networks. We further uncover that online users often form their relations via dual entrainment, while maintain it through single entrainment. Remarkably, the emotions of online users are more convergent in nonreciprocal entrainment. Building on these findings, we develop an entrainment augmented model for emotion prediction. Experimental results suggest that entrainment patterns inform emotion proximity in dyads, and encoding their associations promotes emotion prediction. This work can further help us to understand the underlying dynamic process of large-scale online interactions and make more reasonable decisions regarding emergency situations, epidemic diseases, and political campaigns in cyberspace.

## Introduction

Humans are emotional social beings from birth [1,2]. We transmit various emotional signals to communicate to and influence others. For instance, we usually unify our emotions to resist potential threats (e.g., unauthentic vaccination [3,4], illegal immigration [5], and bad customer experiences [6,7]) or to promote beneficial incidents (e.g., pro-social policies [8] and tobacco cessation [9]). In these scenarios, we always adjust our emotion states according to those of our friends via social interactions. These phenomena are typically conceptualized as entrainment, which was firstly identified by Huygens in 1665, and is generally defined as a tendency for two or more independent rhythmic processes to synchronize with each other [10–12].

Entrainment has been found to be particularly relevant to human emotions, and performs many important social functions. Firstly, emotion entrainment can promote more effective social communications by helping people “feel themselves into” another’s emotional episodes [13–15]. Through this communication process, humans both consciously and unconsciously transmit emotional signals that are essential for fostering social bonds and for maintaining good interpersonal relationships [16–18]. Secondly, emotion entrainment can help cultivate a kind of emotional culture [19]. This functions as a social regulator that calibrates our practical comportment in socialization, and consequently leads to strong group commitments and solidarity. Furthermore, researchers recently uncover that empathy is often connected to entrainment in interpersonal interactions [20–22]. Therefore, the implications of emotion entrainment may promote a nuanced understanding of the processes underlying empathy.

Despite its importance, the principles or patterns of emotion entrainment, to date, are still poorly understood. Most of the existing studies merely explore the entrainment phenomenon in face-to-face interactions based on small-scale or controlled laboratory experiments [18,23,24]. How collective emotion entrains outside of in-person interactions in a large-scale, real world data setting is still unknown. Recently, the proliferation of various online social media platforms has provided entrainment investigation with huge amount of emotion-rich data. In addition, it has been uncovered that emotion cues can also be transferred through these avenues [25–27]. These two facts, together, have laid the groundwork for studying massive emotion entrainment beyond dyads.

However, there are still several other challenges for us to understand the principles of emotion entrainment. Firstly, emotion entrainment on a large scale involves a complex interplay, and often entails dealing with non-linear systems [14]. Existing approaches, both in modeling and analysis, cannot deal with this problem well. Traditional approaches based on various entrainment models are usually computational complex, and the underlying assumptions often violate actual, real-world scenarios [28,29]. While, on the other hand, the more recent network analysis approaches inevitably lack enough detail about entrainment processes [30,31], and often do not distinguish entrainment directions. Secondly, though entrainment phenomena have been investigated from various dimensions (i.e., in-person interactions [32,33], cross-modality communication [23], and social norm calibration [34]), there lacks an effective model that can learn the principles governing entrainment processes well, and predict the future emotions of the targeting individuals or groups effectively.

To deal with the challenges presented above, in this paper, we elaborate a pragmatic framework that can characterize entrainment phenomenon and quantify its patterns on a large scale efficiently. Based on the datasets from large popular social media platforms, we primarily investigate (1) the rules and patterns of emotion entrainment outside of in-person interactions, and then evaluate (2) how different entrainment patterns benefit the prediction of individuals' future emotions. This work can provide significant insights into understanding the underlying dynamic process of large-scale online interactions and make more reasonable decisions regarding emergency situations, epidemic diseases, and political campaigns in cyberspace.

## Results

### Community level entrainment

Previous research has uncovered that massive-scale emotional contagion occurs in online social networks [25]. In this section we further attempt to clarify whether human emotions entrain in social media communities. For our study we use three large-scale, real world datasets, including two English datasets (IR05 [35] and CHI06 [36]) from an emotion sharing blog platform named Livejournal (http://www.livejournal.com/), and one Chinese dataset (Sina Weibo [37]) from a microblogging platform called Sina Weibo (http://www.weibo.com/). The descriptive statistics of the three experimental datasets are summarized in S1 Table. To facilitate emotion entrainment analysis, we conduct polarity classification [38] on these datasets. The 1449 most commonly used mood labels in IR05 and CHI06 datasets are grouped into three emotion states, including positive (POS), neutral (NEU), and negative (NEG) (details are shown in S2 Table). While, for the Sina Weibo dataset, a Naive Bayes classifier [39] is trained to determine the three emotion states. During the computational process, we convert the emotion tags of online users into three discrete values [-1, 0, +1] that characterize negative, neural, and positive emotions respectively. However, an empirical study on emotion entrainment has proven difficult since the entrainment process in observational data is usually confounded by user heterogeneity (e.g., difference in user’s gender, age, and residence) [40,41]. Thus, to further make the results reliable, we have designed a randomized trial by randomly sampling 20k users who have labeled at least 3 emotion tags out of each dataset. As users have been randomly selected, their emotion states differ in expectation only through the entrainment process. With this manipulation, we can independently supervise emotion entrainment in social media communities, while simultaneously minimize user heterogeneity caused by selection bias and data incompleteness [42].

**Fig 1:**
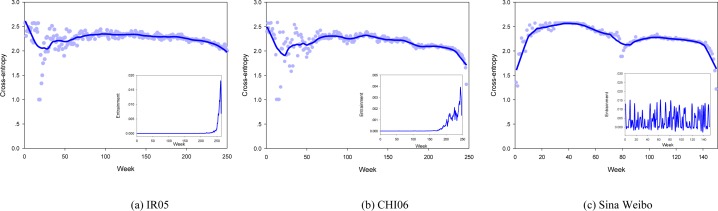
Emotion evolution under entrainment. Inset: entrainment evolution over time.

To understand the evolution patterns of online users’ emotions on the two platforms, we quantify the average emotion distance within a dyad as a function of the community life (Fig 1). Accordingly, we define a variable ${\langle CE\rangle }_{t}=\frac{1}{N^{2}}\sum_{i\neq j}CE\left(p\left(v_{i}^{\;t}\right),p\left(v_{j}^{\;t}\right)\right)$ to measure the emotional distance between two users, where *p*(*v*_*i*_^*t*^) and *p*(*v*_*j*_^*t*^) respectively represent the probability distribution of emotion states for user *v*_*i*_ and user *v*_*j*_ at timestamp *t*, while CE(*) calculates the cross-entropy [43] between them. Smaller 〈*CE*〉_*t*_ value indicates shorter emotional distance between the two users. To supervise how emotional distance changes over entrainment process, we utilize *Etr*(*v*_*i*_ → *v*_*j*_) = *TE*(*v*_*j*_ → *v*_*i*_) to measure entrainment strength from user *v*_*i*_ to user *v*_*j*_ based on transfer entropy (TE) [44]. Different from previous work, the proposed entrainment measure in this paper, which is asymmetric and allows differentiation in the direction of entrainment, can capture complex emotion dynamics without modeling the exact interactions within dyads. In addition, unlike existing studies that are almost concerned with aggregate measures for entrainment quantification [23,32], the measure presented in this paper allows more fine-grained characterization on the information flow among individuals. This key property enables us not only to study massive emotion entrainment at the community level, but also to examine different entrainment patterns at the peer level. For more details of the measurement, please refer to the Materials and Methods section.

Fig 1 suggests that emotion entrainment occurs (drops in cross-entropy value) as the communities develop. At the inception of Livejournal, users in the community are emotionally dissimilar. As entrainment strength enhances (Insets of Fig 1(A) and 1(B)), emotion disparity among users decreases (blue curves in Fig 1(A) and 1(B)). In contrast, users in Sina Weibo experience a quite different entrainment process. Primarily, users are emotionally similar to each other. This anomalous phenomenon may be attributed to the time delay in data collection (about 5 months after the public release of Sina Weibo platform), which allows users on the platform to entrain for a certain period of time before our surpervision. Hereafter, collective emotions in Sina Weibo roughly undergo two entrainment cycles (Fig 1(C)). During this process, entrainment strength vibrates rhythmically (Inset of Fig 1(C)), which causes the entrainment phenomenon transient without entering a stable state.

**Fig 2:**
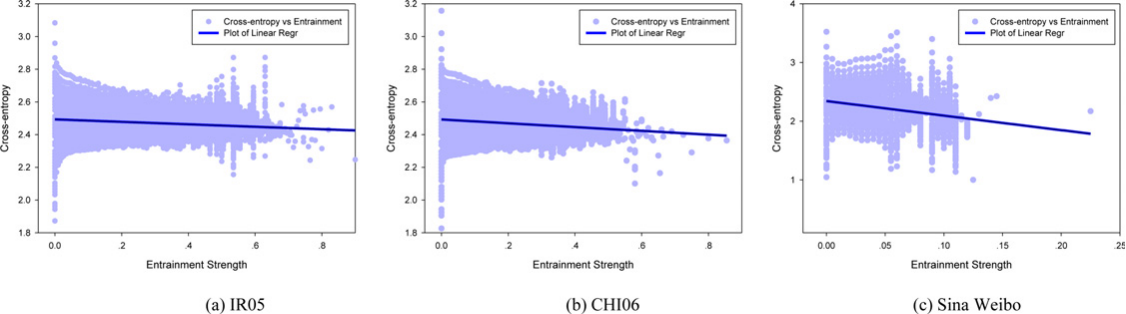
Emotional distance with respect to entrainment strength (circles). Regression lines are colored in blue, with coefficients of -0.08, -0.12 and -2.48 respectively for (a) (b) and (c).

As entrainment promotes rapport and social closeness [45], we subsequently turn to examine how emotions evolve as entrainment strength enhances. Specifically, we consider the emotional distance 〈*CE*〉_*T*_ as a function of the minor value of reciprocal entrainment strength (Fig 2). Here, T is the total observation period in a dataset.

We observe that as entrainment strengthens, the average emotional distance decreases (blue regression lines in Fig 2). This tendency indicates that users are more emotionally similar to each other under stronger entrainment process. Compared with that of Livejournal, the emotional distance in Sina Weibo drops more sharply as entrainment strength enhances (Fig 2(C), with the lowest regression coefficient of -2.48). Notably, this entrainment process develops in a moderate way, with the maximum entrainment strength value no more than 0.25. This observation is inline with previous contention that moderately rhythmic social interactions generally promote social closeness and positive experience [46].

### Peer level entrainment

Understanding how social relationship and emotion interact may require further close scrutiny in peer level entrainment. Since whether emotion entrainment is directional or not is unclear yet, previous work simply eludes this issue by measuring entrainment in a symmetric way [23,47]. In this section, we primarily clarify this issue by probing the reciprocal entrainment strength within each user pair. If the strength of reciprocal entrainment differs significantly, then emotion entrainment can be considered as directional. Otherwise, it can be regarded as undirectional. Under this assumption, we introduce a variable *EnDis* to quantify entrainment difference for a given user pair *v*_*i*_ and *v*_*j*_:

$$EnDis\left(v_{i},v_{j}\right)=\frac{abs\left(Etr\left(v_{i}\to v_{j}\right)-Etr\left(v_{j}\to v_{i}\right)\right)}{max\left(Etr\left(v_{i}\to v_{j}\right),Etr\left(v_{j}\to v_{i}\right)\right)}$$ (1)

where, abs(*) and max(*) respectively calculate the absolute value and the maximum value of the function enclosed.

**Fig 3:**
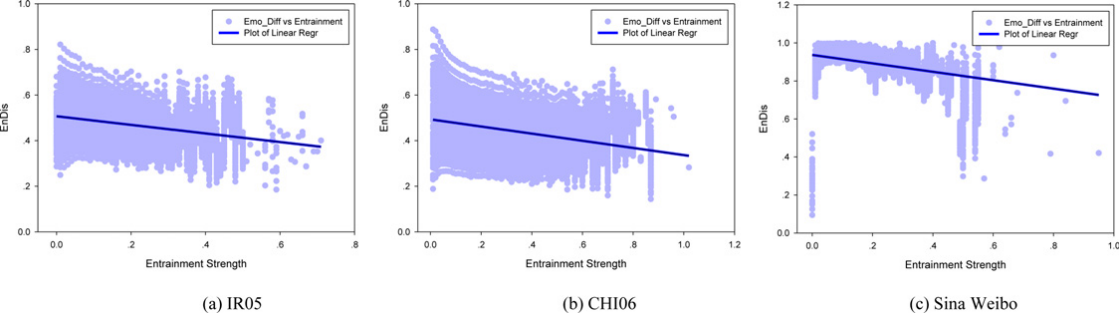
Disparity in entrainment strength within each user pair. Here, *EnDis* is considered as a function over entrainment strength. Average *EnDis* in (a), (b), (c) are 0.431, 0.439, 0.828 respectively.

Given this definition, we calculate *EnDis* for each user pair and further show how *it* changes over entrainment strength (Fig 3). Experimental results indicate that there is significant disparity in reciprocal entrainment strength within dyads. The average *EnDis* values of IR05, CHI06, and Sina Weibo are 0.431, 0.439, and 0.828 respectively. These results demonstrate that emotion entrainment on these social media platforms is directional. Notably, this phenomenon is more significant in Sina Weibo, which indicates that single entrainment is more pervasive on this platform.

Further, from Fig 3, we find that there exists an uncannily correlation between *EnDis* and entrainment strength, i.e., *EnDis* decreases as entrainment strength increases (denoted by the blue regression lines in Fig 3). This observation implies a positive correlation in reciprocal entrainment, i.e., if an individual entrains strongly towards his interaction partner, then his partner is likely to entrain back in a similar way. The corresponding Pearson's Correlation Coefficients (PCCs) are 0.737, 0.728, and 0.192 respectively in Fig 3(A), 3(B) and 3(C) (p<0.0001). The lowest correlation coefficient in Sina Weibo implies that users on this platform more often entrain in single way. It has been revealed that social contacts are tied to intimate relationships that often assume the same or similar emotions [48]. Thus, the positive correlation in dyadic entrainment may manifest that users bearing strong reciprocal entrainment relationship are more emotionally similar. Definitive answer for this issue necessitates delving deeper into peer level entrainment. To this end, at each timestamp *t*, we divided all user peers into three groups:

$$\text{G}\left(v_{i}{}^{t},v_{j}{}^{t}\right)=\left\{ \begin{array}{l} \left\{\text{Dual}|Etr\left(v_{i}{}^{t}\to v_{j}{}^{t}\right)\geq \theta_{t}\wedge Etr\left(v_{j}{}^{t}\to v_{i}{}^{t}\right)\geq \theta_{t}\right\},\\ \left\{\text{Single}|\left(Etr\left(v_{i}{}^{t}\to v_{j}{}^{t}\right)\geq \theta_{t}\wedge Etr\left(v_{j}{}^{t}\to v_{i}{}^{t}\right)<\theta_{t}\right)\vee \left(Etr\left(v_{i}{}^{t}\to v_{j}{}^{t}\right)<\theta_{t}\wedge Etr\left(v_{j}{}^{t}\to v_{i}{}^{t}\right)\geq \theta_{t}\right)\right\},\\ \left\{\text{None}|Etr\left(v_{i}{}^{t}\to v_{j}{}^{t}\right)<\theta_{t}\wedge Etr\left(v_{j}{}^{t}\to v_{i}{}^{t}\right)<\theta_{t}\right\}. \end{array}\right.$$ (2)

where, users in the group Dual and in the group Single respectively entrain in dual way and in single way, while users in the group None do not entrain at all. *θ*_t_ represents the average entrainment strength at timestamp *t*. This time-dependent division for entrainment patterns is imperative. Previous research suggests that tight entrainment process does not always guarantee good social experiences, while moderately rhythmic social interactions generally provide a more positive result [46]. Therefore, it is unreasonable to simply use a unified threshold to distinguish different entrainment patterns. Comparatively moderate entrainment process at the current timestamp, though below a predefined threshold, may promote more efficient emotional interactions at other timestamps. As such, we examine entrainment patterns at the peer level with time-varying thresholds. In what follows, without ambiguity, we omit the superscript *t* (e.g., simplifying G(*v*_*i*_^*t*^, *v*_*j*_^*t*^) to G(*v*_*i*_, *v*_*j*_)) to indicate a variable at timestamp *t*.

**Fig 4:**
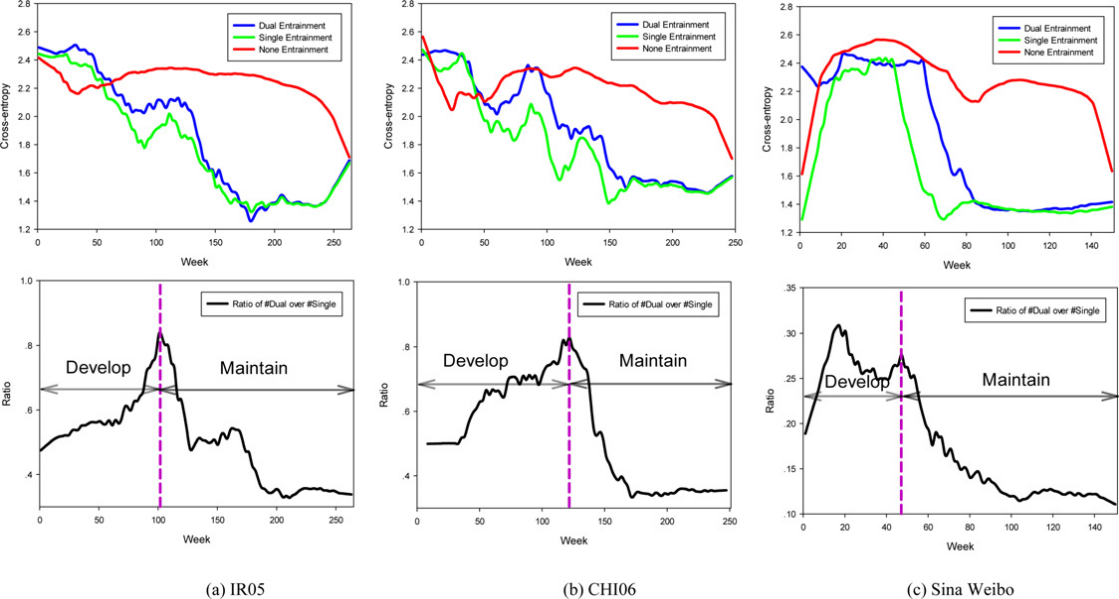
Emotional distance at different peer levels (Top figures). Bottom figures correspond to the varying ratio of the number of Dual entrainment over the number of Single entrainment; purple dashed lines separate the two stages of relationship establishment: ‘Develop’ stage and ‘Maintain’ stage.

Given the group division according to Eq 2, we discern how users’ emotions evolve at different peer levels (top figures in Fig 4). Experimental results indicate that emotions are different for those who do not entrain to each other (p<<0.001 according to an independent two-tailed t-test). These results mean that users’ emotions tend to converge under the entrainment process. Also, we find that emotion distance in the group Dual is larger than that in the group Single at most of the time, though such difference diminishes gradually as entrainment process proceeds (after about two thirds of the total lifespan of the community). This finding appears to be contradictory since we previously reveal that reciprocal entrainment is associated with high emotion proximity.

According to our analysis, the higher emotional difference in the Dual entrainment group can be explained by two competing hypotheses:

1. Users mutually entrain when they are emotionally dissimilar, while switch to single entrainment once they become emotionally familiar.
2. Users become emotionally similar through single entrainment, and afterwards they undergo mutual entrainment in a loose manner to sustain their relationships.

**Fig 5:**
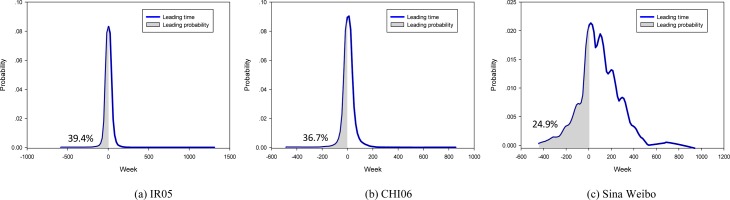
Distribution of leading time of Dual entrainment over Single entrainment.

In order to tease these two hypotheses apart, we examine the probability that users experience dual entrainment ahead of single entrainment (Fig 5). Results suggest that users on Livejournal and Sina Weibo often entrain in single way initially (p = 60.6–75.1%). In Livejournal, single entrainment merely leads weakly ahead, and the time difference (leading or lagging) is relatively small for most of the whole process (as indicated by the sharp peak around zero week in Fig 5(A) and 5(B)). This means users in Livejournal experience relatively short-lived Single entrainment procedure, and quickly switches to Dual entrainment process. The pervasion of Dual entrainment in Livejournal can be considered as a reflection of its users' extroversion in online socialization, as has been found in other social media platforms [49–52]. In contrast, users in Sina Weibo more often experience Single entrainment initially (75.1%). Further, the leading time of this process over Dual entrainment is more smoothly distributed (Fig 5(C)). This compound fact indicates that emotion interactions on this platform necessitate more proactive engagement.

To gain further insights into how Dual entrainment and Single entrainment impact users’ emotions, we examine the relative ratio between these two processes and supervise how it changes along with emotion evolution. At each timestamp *t*, we define a variable $R_{t}=\frac{\#Dual_{t}}{\#Single_{t}}$, where #*Dual*_*t*_ and #*Single*_*t*_ stand for the number of user pairs engaging in Dual entrainment and Single entrainment respectively (bottom figures in Fig 4).

From Fig 4, we can find that despite having different lifespans, peer entrainments on the two platforms all follow a determined two-stage process: an increase in dual entrainment followed by a decrease trend. As *R*_*t*_ approaches its peak, the emotional distance in dyads decreases continuously (in Sina Weibo, the emotional distance decreases after reaching the second crest). We conjecture that this process corresponds to the relationship establishment stage, where emotion proximity develops in dyads. After the peak, entrainment switches to proceed in single way and the emotional distance further decreases. This indicates that users begin to sustain their established relationships. During this process, we notice that entrainment strength in Dual entrainment process is weaker than that in Single manner (p<0.05 according to an independent one-tailed t-test). This finding implies that moderate entrainment is more beneficial in establishing social relationships, yet sustaining it warrants more endeavor. According to these findings, we surmise that the establishment of online social relationships typically undergoes three entrainment stages, i.e., Single-Dual-Single. Originally, relationship establishes with an individual proactively entrains towards another (Single entrainment); then the relationship develops via mutual adaption in users’ emotions (Dual entrainment); finally, it sustains mainly in one way entrainment (Single entrainment).

Another meaningful finding we obtained from Fig 4 is that the time of fulfilled Dual entrainment (i.e., the second stage) is not fixed on different platforms. In Livejournal, it takes about half of the community lifespan to undergo Dual entrainment (i.e., establishing social relationships). In contrast, users in Sina Weibo experienced a relative short period of Dual entrainment process (about one third of the community lifespan) before morphing into Single entrainment procedure. In addition, users in Sina Weibo are more emotionally similar at the end of the entrainment process. These facts together ascertain that the establishment of social relationship in Sina Weibo is more efficient.

The above observations suggest that entrainment patterns in dyads are possible to inform something about their emotion proximity. For instance, if a user pair enters Single entrainment after experiencing Dual entrainment process for long, these pairwise users are very likely to have established an intimate social relationship, and they may assume similar emotions in the near future. This knowledge permits a better surpervision and prediction on the emotion evolution of online communities. This issue, if well tackled, may help to prevent potential negative effects incurred by emergency situations [53,54], epidemic diseases [3], and political campaigns [8,55,56]. In what follows we will harness these insights in the setting of an emotion prediction task.

### Emotion Prediction

By analyzing emotion entrainment in social media both at the community level and the peer level, we have uncovered some key principles governing the interactions between entrainment and emotion dynamics. We turn now to showing that these principles are predictive of emotional proximity within dyads, and could be used to leverage emotion prediction. Since online communities manifest stronger emotions than face-to-face interactions [57], emotion prediction can help us to understand online communications and further take corresponding counteractions timely. Also, emotion prediction can promote social coherence [58,59] by identifying “isolated” users early and recruit them in the community.

**Fig 6:**
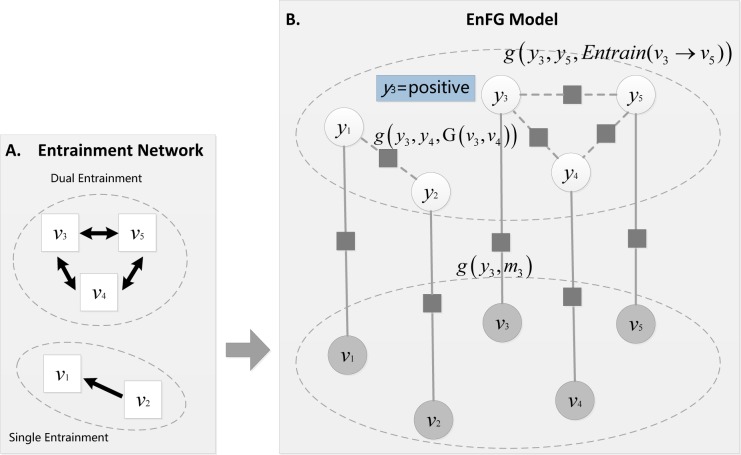
Graphical representation of the EnFG model. Panel A: entrainment network constitutes Dual entrainment and Single entrainment patterns; Panel B: EnFG model based on entrainment network.

Most traditional classifiers learn and make predictions on each sample in isolation. However, modeling proximity relationships between samples enables us to leverage *coherence* [60–63], i.e., similar samples may share the same status. Entrainment network delivers insights into understanding social relationships (Fig 6 Panel A), yet existing work is incapable of providing a flexible way to encode such information. Thus, we elaborate an entrainment augmented factor graph model (hereafter EnFG model) to encode entrainment information and all other features into a unified framework. This model establishes relationships between entrainment information and user emotions by defining various factor functions, and then makes tractable inference via factorizing the “global” probability as a product of such “local” functions ((Fig 6 Panel B)). By elaborating the EnFG model, we show how entrainment information informs users' future emotions. This modeling framework can also be readily generalized to other emotion related tasks, such as relationship identification [18,64], social stratification [65], and affiliative community detection [66,67].

The proposed EnFG model is formulized as:

$$ο\left(\theta \right)=\sum_{i=1}^{N}\sum_{j=1}^{d}\alpha_{j}g\left(y_{i},m_{ij}\right)+\sum_{e_{ij}\in E}\beta_{ij}g\left(Etr(v_{i}\to v_{j})\right)+\sum_{e_{ij}\in E}\gamma_{ij}g\left(\text{G}\left(v_{i},v_{j}\right)\right)-\text{log}\;Z$$ (3)

This model involves three factor functions, including the modality factor *g*(*y*_*i*_, *m*_*ij*_), the entrainment association factor *g*(*Etr*(*v*_*i*_ → *v*_*j*_)), and the entrainment pattern factor *g*(G(*v*_*i*_, *v*_*j*_)). *g*(*y*_*i*_, *m*_*ij*_) captures the correlation between the user *v*_*i*_’s j^th^ modality *m*_*ij*_ and his emotion state *y*_*i*_, which is defined as:

$$g\left(y_{i},m_{ij}\right)=\left\{ \begin{array}{l} 1,\;if\;m_{ij}\;occurs\;with\;y_{i},\\ 0,\;otherwise. \end{array}\right.$$ (4)

where, *m* is the modality matrix, whose element *m*_*ij*_ correspods to the j^th^ attribute of user *v*_*i*_. Previous research reveals that human emotions have memories [68] and are partly reflected by user’s collective behaviors [69–71]. Thus, when define the modality factor function, we mainly utilize two kinds of modalities: historical emotions and activity level. For the sake of efficiency and simplicity, we employ the n-gram models [72] to encode historical emotions. In this modeling scheme, we use unigrams (i.e., encoding each emotion state separately) to depict user emotion at each timestamp, while use bigrams (i.e., encoding two adjacent emotion states as a whole) to capture emotion dependency across different timestamps. In measuring user's activity level, we count the total amount of messages one individual posted at each timestamp. Considering the feature sparsity problem [52,73], we discretize the activity level of online users into three intervals: low, medium, and high. Thresholds separating the intervals are set respectively as one time and two times of the average posting number. At the same time, the activity level is also encoded with unigrams and bigrams. Finally, we differentiate users’ emotional and behavioral impacts occurring at distinct timestamps by combining each tag with its relative temporal index. All these features and those defined below are summarized in S3 Table.

*g*(*Etr*(*v*_*i*_ → *v*_*j*_)) depicts whether user *v*_*i*_ ∈ *V* entrains to user *v*_*j*_ ∈ *V*. This function is given by:

$$g\left(Etr(v_{i}\to v_{j})\right)=\left\{ \begin{array}{l} 1,\;if\;Etr(v_{i}\to v_{j})\geq En_{0},\\ 0,\;otherwise. \end{array}\right.$$ (5)

where, *En*_0_ is set as the average entrainment strength in the past seven days over all user pairs.

*g*(G(*v*_*i*_, *v*_*j*_)) characterizes the entrainment pattern between users *v*_*i*_ ∈ *V* and *v*_*j*_ ∈ *V*, and is defined as:

$$g\left(\text{G}\left(v_{i},v_{j}\right)\right)=\left\{ \begin{array}{l} 2,\;if\;\left[\text{G}\left(v_{i},v_{j}\right)==Dual\right],\\ 1,\;if\;\left[\text{G}\left(v_{i},v_{j}\right)==Single\right],\\ 0,\;otherwise. \end{array}\right.$$ (6)

where, G(*v*_*i*_, *v*_*j*_) is given by Eq 2.

*α*, *β*, and *γ* are respectively weights of the three different factor functions; **θ** = (*α*, *β*, *γ*) is a parameter configuration estimated from the training data; and *Z* is a normalization factor to ensure that the distribution is normalized so that the sum of the probabilities equal to 1. An example of the EnFG model is illustrated in Fig 6 Panel B.

To evaluate the proposed EnFG model, in each dataset (IR05, CHI06, and Sina Weibo), we select the top 10K users who have posted the most emotion tags to constitute the evaluation pool. In our experiments, we choose users’ historic modalities (i.e., emotion tags and activity levels) as the training data and use the learned model to predict their respective emotion state (i.e., positive, neutral, and negative) in the final timestamp. In the following experiment, each day is considered as a timestamp. For evaluation metric, we use prediction accuracy. All the following experiments are the average four-fold cross-validation results.

To clarify how EnFG performs compared with traditional approaches, we also employ several alternative approaches, including Naïve Bayes (NB), Maximum Entropy (ME) [74], Support Vector Machines (SVMs) [75], and Radial Basis Function Networks (RBFN) [76]. Essentially, these comparison approaches do not provide a flexible way to capture association information. As such, to characterize Dual entrainment information, we group users through a chain clustering procedure. We then consider two users belong to the same group according to:

$$\text{SG}\left(v_{i},v_{j}\right)=\left\{ \begin{array}{l} 1,\;if\;Etr\left(v_{i}\to v_{j}\right)\geq En_{0}\wedge Etr\left(v_{j}\to v_{i}\right)\geq En_{0},\\ 0,\;otherwise. \end{array}\right.$$ (7)

where, *En*_*0*_ is a similarity threshold.

To encode Single entrainment relationship in the comparison approaches, for each user *v*_*i*_, we select out his top K (= 5) most solely entrained neighbors (*v*_*j*_) and average their emotion distributions (**d**_*j*_) by weighting with the corresponding entrainment strength:

$$D_{i}=\frac{\sum_{j=1}^{K}Etr\left(v_{i}\to v_{j}\right)\cdot d_{j}}{\sum_{j=1}^{K}Etr\left(v_{i}\to v_{j}\right)}$$ (8)

where, each dimension in **d**_*j*_ corresponds to one emotion state, i.e., positive, negative or neutral.

Considering the data sparsity problem, Eq 8 is further discretized as:

$$f\left(D_{i}\left[l\right]\right)=\left\{ \begin{array}{l} 1,\;if\;D_{i}\left[l\right]\in [0,1/4),\\ 2,\;if\;D_{i}\left[l\right]\in [1/4,1/2),\\ 3,\;otherwise. \end{array}\right.$$ (9)

This entrainment modulated distribution is assumed to suggest the tendency of emotion evolution for each user.

**Fig 7:**
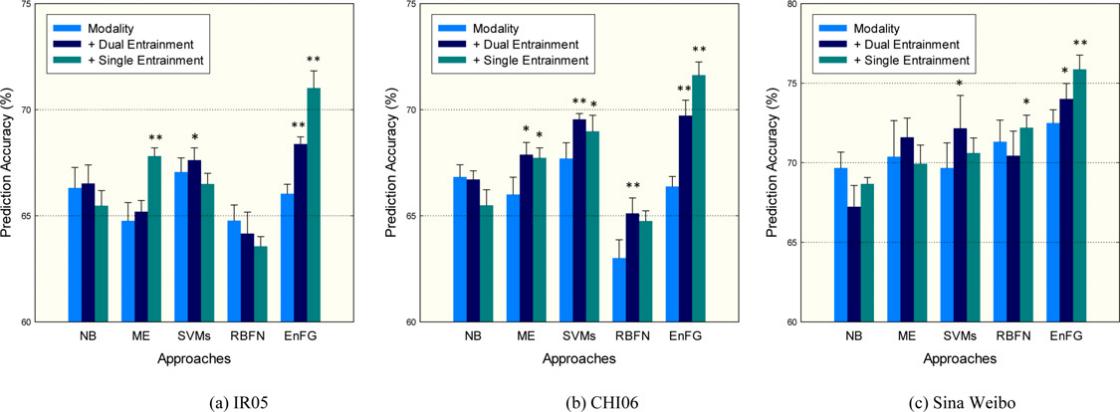
Prediction performances when different peer entrainments are added sequentially. Significance of performance improvement over modality features according to a paired two-tailed t-test is indicated using *-notation (* = "p<0.05",** = "p<0.01").

Fig 7 represents the evaluation results for all approaches on the evaluation datasets. Experimental results suggest that entrainment information generally benefits emotion prediction task, albeit with a few exceptions. The performance gain may be attributed to the associations established between emotional similarity and different entrainment patterns. Compared with other two datasets, Sina Weibo dataset is confirmed to be the most predictable (3.77–11.47% higher in performance). We conjecture this fact is due to the higher efficiency in entrainment process in Sina Weibo, which engenders more coherence information to be leveraged in prediction. While, Dual entrainment information has more significant prediction impacts on IR05 and CHI06 (respectively 66.04% and 418.87% higher improvement than that in Sina Weibo). This suggests that entrainment plays an important role in social networks where users' emotions are publicly sensible to each other.

Another experimental results indicate that distinguishing different entrainment patterns (Dual entrainment and Single entrainment) can further benefit prediction performance. While Dual entrainment information benefits most approaches employed in this experiment, Single entrainment consistently boost performance in our proposed EnFG model. Though the EnFG model does not perform well with mere modality features, it does predict user emotion with high accuracy by characterizing different peer entrainment information. In terms of accuracy, EnFG achieves a 3.84–5.08% higher performance compared with the four alternative approaches encoding the same feature sets. By incorporating entrainment information, EnFG boosts the original model with only modalities by 4.66–7.91% in precision. These results validate that EnFG model is both efficient and effective in characterizing entrainment information.

## Discussion

In this paper, we explore emotion entrainment in the context of two large social media platforms, including Livejournal and Sina Weibo. We study the emotion entrainment phenomenon at both the community level and the peer level. When examining the evolution of massive emotions on the two platforms, we find that collective emotions entrain with the evolution of communities. Especially, users' emotions in Sina Weibo roughly undergoes two entrainment cycles. During this process, entrainment strength vibrates rhythmically, making the entrainment phenomenon transient without entering a stable state. Additionally, we find that users become emotionally similar as entrainment enhances. This tendency indicates that users are more emotionally similar to each other under stronger entrainment process.

To discern entrainment closely, we examine entrainment at the peer level. We find that difference in reciprocal entrainment strength is significant on both platforms, indicating that emotion entrainment is directional. Specifically, this difference is most significant in Sina Weibo, which means users are customary to entrain asymmetrically on this platform. Also, we obtain that there exists a positive correlation in reciprocal entrainment. Since social contact is closely tied to emotional proximity, we are motivated to discern how emotions entrain in different peer patterns, i.e., Dual entrainment, Single entrainment and None entrainment. By studying the emotion evolution under different peer levels, we uncover that emotion difference is significant in dyads with no entrainment. In addition, we reveal that emotion difference in Dual entrainment is larger than that in Single entrainment. These findings indicate that users entraining in single way are more emotionally similar. To explain this superficially contradictory phenomenon, we calculate the relative time lag between Dual entrainment and Single entrainment and surprise the temporal ratio between them. Experimental results imply that the establishment of social relationship undergoes three stages, i.e., it launches with one individual proactively entrain towards his interaction partner, then develops mutually, and finally maintains mainly in single way. This finding is remarkable since it informs users’ future emotions through the lens of entrainment patterns. Although randomized trials are adopted in these analyses, it is notable that online user emotions may also be influenced by exogenous factors such as cross-platform interferes and unobservable offiline interactions. In these scenarios, our entrainment measurement are able to work. The proposed measurement is constructed based on transfer entropy that is sensitive to all order correlations. Furthermore, it aims to capture the underlying causation between two users rather than correlation. Therefore, this measurement setting is likely to gurantee relatively relabible results in open social systems.

Based on previous empirical findings, we then construct an entrainment augmented computational model for predicting user emotions. Different from existing work, our model provides a flexible way to encode different entrainment patterns by defining various factor functions. Experimental results prove that the proposed model is both efficient and effective in characterizing entrainment information. Furthermore, this modeling framework has practical implications by suggesting a possible way to encode entrainment information, and can be readily generalized to other emotion related analysis. What’s more, this work can help us to understand the underlying dynamic process of large-scale online interactions and make more reasonable decisions regarding emergency situations, epidemic diseases, and political campaigns in cyberspace.

## Materials and Methods

### Materials

Livejournal is a social media platform where users share passions and opinions under various kinds of topics. Aside from posting messages, this platform also allow users to label their moods. We suppose these mood tags correspond to users’ temporary emotion states, which are sufficient proxies for us to analyze the entrainment patterns. On this platform, IR05 spans a period of 5 years—from 2000 until 2005, and consists of 33K users and 624K mood tags in total. Also, it is emotional rich, with each user has published 24 posts and labeled 18 mood tags in average. CHI06 is an alternative dataset collected from Livejournal, which contains approximately 18 million English blog posts written by about 1.6 million bloggers. In average, each user writes 10 posts and labels 6 emotion tags. This dataset covers a 48 months period from 1st May 2001 to 23rd Apr. 2005. On the other hand, Sina Weibo is a Twitter-like microblogging system in China. With more than 40 million active users spreading approximately 100 million messages each day, this system is generally considered as an ideal laboratory for studying Chinese content. The Sina Weibo dataset contains 1.7 million users who generate about 12 million posts. The time span in this dataset is from 15th Jan. 2010 to 27th Nov. 2012. These three long period emotional rich datasets present us with an unprecedented opportunity to study emotion entrainment over users’ entire lifespans.

**Fig 8:**
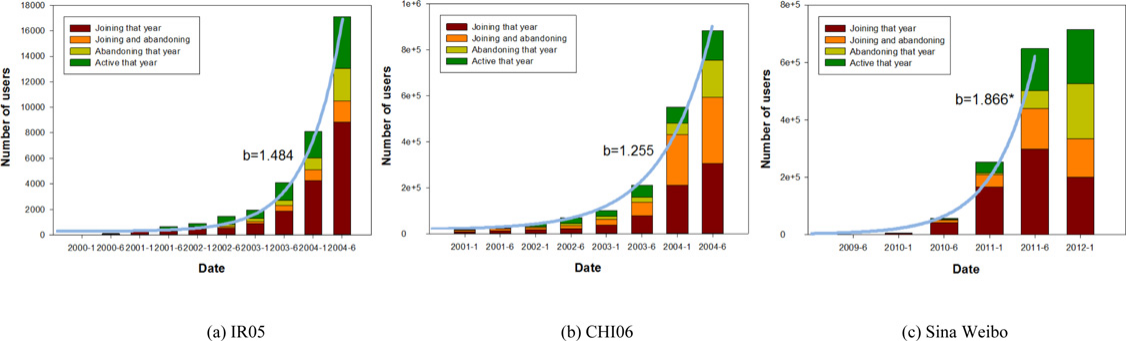
Change in user base. Breakdown of active users each year. From bottom up: users that joined the community that year (and did not abandon the same year), users that joined and abandoned the community that year, users that abandoned the community that year (and did not join the same year) and other active users. Blue curve corresponds to fitted exponential function: y = a*exp(b*(t-t_0_)). ‘*’ means only partial data samples are used in curve fitting.

**Fig 9:**
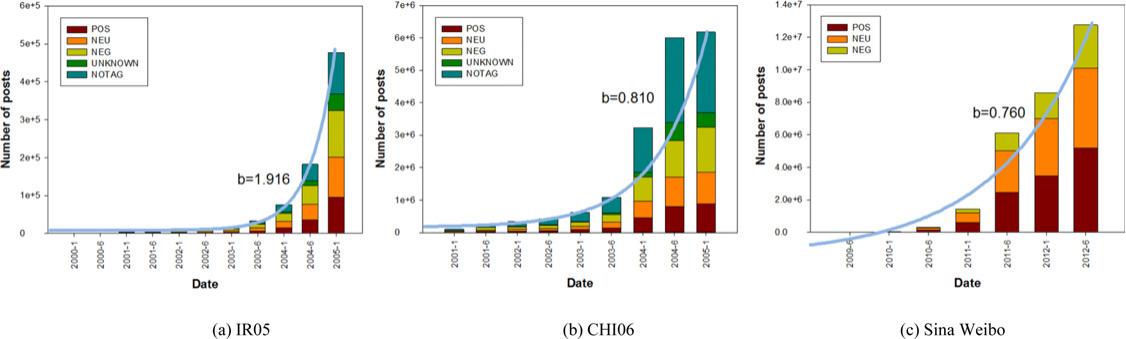
Change in posting number. Breakdown of posts each year. From bottom up: posts with positive emotion tags (POS), neutral emotion tags (NEU), negative emotion tags (NEG), unknown emotion tags within our emotion classification scheme (UNKNONWN), and no emotion tags (NOTAG). Blue curve corresponds to fitted exponential function: y = a*exp(b*(t-t_0_)).

For the two platforms, including Livejournal and Sina Weibo, we consider that a user joins the online community after publishing his first post, and abandons it if he does not contribute any post for at least six months. To enforce this policy, in depicting the change in user base, we ignore users that have posted within the last six months in each community. Fig 8 shows a breakdown of active users showing the number of users joining and abandoning their communities over time. We find that the growth of user number follows by the exponential function (blue curves in Fig 8). In Sina Weibo dataset, however, the assumption for exponential growth only holds exclusive of data samples in the last half year (i.e., 2012–1 in Fig 8(C)). This fact is due to the cessation in exponential growth in user numbers, as reflected by the large portion of users abandoning the community in the last half year. Fig 9 illustrates the change in posting number which are divided into five groups, i.e., posts with positive emotion tags (POS), neutral emotion tags (NEU), negative emotion tags (NEG), unknown emotion tags within our emotion classification scheme (UNKNONWN), and no emotion tags (NOTAG). The result shows that the total number of posts also grows exponentially (blue curves in Fig 9).

To protect user privacy, in our study, all datasets are anonymized by substituting real user ids with random number references. In Sina Weibo dataset, there is no emotion label for each post. Therefore, we have trained a Naive Bayes classifier to determine positive, negative or neutral polarity for each post. The training dataset [77] contains over 3.5 million labeled messages written by approximately 7K users from Sina Weibo. In designing the classifier, we choose to use unigrams and bigrams features since they proves to be effective in this dataset. Also, we take into account negation rules by adding a prefix ‘neg-’ for all features modified by a negation indicator [78]. To tackle with polarity shifting [79,80] and the emergences of neologisms [81,82], a self-learning scheme [83] is implemented in the classifier. This scheme gradually enhances the trained classifier by augmenting training dataset with testing samples assigned with high prediction confidence. Overall, this classifier achieves an empirical precision of 72.18% with four-fold cross-validation on the dataset.

### Methods

To construct the measurement framework for emotion entrainment, we mainly take two aspects into consideration. First, to make the framework reasonable and scalable for large datasets, we should assume as few hypotheses as possible. Second, whether emotion entrainment is directional or not is unclear yet, we should design the measurement asymmetrically to distinguish entrainment direction, and then validate its rationality with corroborating experimental evidence. As such, we adopt transfer entropy to guide the design of entrainment measure since this approach is asymmetric and can capture arbitrary nonlinear interactions well. As human emotions are found to have certain narrative memories [68], we can make predictions for each individual’s future emotions based solely on part of his emotion history. This means online user emotions satisfy the Markov property [84], and we can represent the history of emotions for each user x by a Markov process X = xt (S1 Fig). According to Schreiber [85], the transfer entropy from users y to x is given by:

$$TE\left(\text{Y}\;\to \;\text{X}\right)=H\left(x_{t+1}|x_{t}^{m}\right)-H\left(x_{t+1}|x_{t}^{m},y_{t}^{n}\right)$$ (10)

where, $x_{t}^{m}=\left(x_{t},\ldots ,x_{t-m+1}\right)$, $y_{t}^{n}=\left(y_{t},\ldots ,y_{t-n+1}\right)$, while *m* and *n* are the orders (memory) of the Markov process *X* and *Y* respectively. For the sake of simplicity, we take *m = n = 3* from this point on. According to our empirical analysis, this order is high enough, since higher order merely increases computational cost without present much quantitative differences. H(*) calculates entropy over a given probability distribution.

Eq 10 amounts to the uncertainty reduction by using the historical emotions of user y to predict the next emotion state of user x. This capacity to predict user x due to the knowledge of user y reflects the potential that user x adopts his emotions towards that of user y. Thus, the entrainment strength from user x to user y at the emotion level can be quantified as:

Etr(X→Y)=TE(Y→X) (11)

Estimating Eq 11 based on finite data is prone to incur biases and statistical errors [86,87]. As such, we choose to use Simpson’s rule [88] as the entropy estimator, which gives more accurate approximation for entrainment strength *Etr*(X → Y) via piecewise quadratic. In what follows, we present the complexity analysis for this estimation procedure. According to Simpson’s rule, Eq 11 can be approximated by calculating the following composite Simpson quadrature:

$$Etr=\int_{a}^{b}Etr^{'}\left(x\right)\;dx\approx \frac{h}{3}\left[Etr^{'}\left(x_{0}\right)+2\sum_{i=1}^{n/2}Etr^{'}\left(x_{2i-2}\right)+4\sum_{i=1}^{n/2}Etr^{'}\left(x_{2i-1}\right)+Etr^{'}\left(x_{n}\right)\right]$$ (12)

where, the integration interval [a, b] is divided into *n* even subintervals (or grids), *x*_*i*_ = *a* + *ih*, 0 ≤ *i* ≤ *n*, $h=\frac{b-a}{n}$, and *Etr*'(*x*) corresponds to the derivation of function *Etr(x)*. This computation is efficient with a complexity of *O*(*N* log *N*), where *N* equals to the number of grid points. This cost is acceptable in our datasets.

## Supporting Information

S1 Fig Illustration of entrainment quantification. (PDF)

S1 Table Data Statistics. (PDF)

S2 Table Categories of emotion tags. (PDF)

S3 Table Features Used in Emotion Prediction. (PDF)
