# Supplementary material for: Exploring Entrainment Patterns of Human Emotion in Social Media
Source: PLoS One. 2016 Mar 8;11(3):e0150630. doi: 10.1371/journal.pone.0150630 (PMC4782991; doi:10.1371/journal.pone.0150630)
Supplement: S1 Fig — (PDF) [file pone.0150630.s001.pdf]

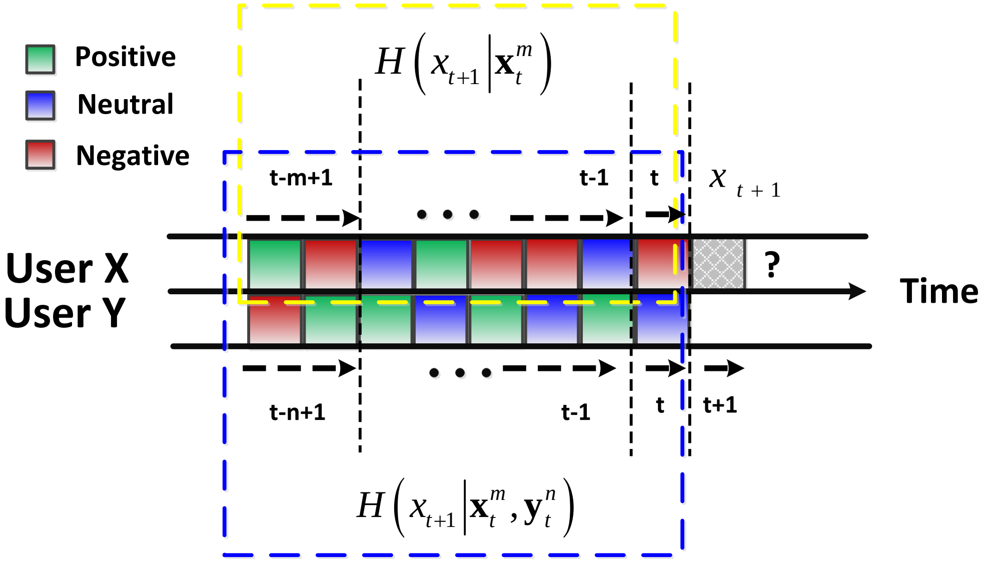

S1 Fig. Illustration of entrainment quantification. $H\left(x_{t+1}\middle|\mathbf{x}_t^m\right)$ amounts to the uncertainty about user *x*, $H\left(x_{t+1}\middle|\mathbf{x}_t^m,\mathbf{y}_t^n\right)$ amounts to the uncertainty about user *x*, if we know the emotions of user *y*.
