# Supplementary material for: Exploring Entrainment Patterns of Human Emotion in Social Media
Source: PLoS One. 2016 Mar 8;11(3):e0150630. doi: 10.1371/journal.pone.0150630 (PMC4782991; doi:10.1371/journal.pone.0150630)
Supplement: S1 Table — (PDF) [file pone.0150630.s002.pdf]

Table 1. Data statistics.

Item		IR05		CHI06*	Sina Weibo
Number of	posts	815,494	(24.26/user)	18,235,056 (10.86/user)	24,055,810 (13.54/user)
Number of	emotion tags	624,905	(18.59/user)	10,521,161 (6.27/user)	--
Number of	users	33,611		1,678,810	1,776,950
Number of	Months	60		48	34

Note: ‘--’ means unavailable. *: for CHI06 dataset, the statistics is conducted only on English messages.
