# Supplementary material for: Exploring Entrainment Patterns of Human Emotion in Social Media
Source: PLoS One. 2016 Mar 8;11(3):e0150630. doi: 10.1371/journal.pone.0150630 (PMC4782991; doi:10.1371/journal.pone.0150630)
Supplement: S2 Table — (PDF) [file pone.0150630.s003.pdf]

Table 2. Categories of emotion tags.

Category	Examples of emotion tags	Sample Number
Positive	great, elated, cheerful, ecstatic, jovial, fantastic, whee, triumphant, perky, …	285
Neutral	calm, so so, at peace, normal, ready for bed, working, thirsty, busy, blah, snuffly, warm, alive, …	665
Negative	bored, sore, depressed, homicidal, crappy, yucky, remorseful, bitchy, befuddled, edgy, …	499
Total		1449
