# Supplementary material for: Exploring Entrainment Patterns of Human Emotion in Social Media
Source: PLoS One. 2016 Mar 8;11(3):e0150630. doi: 10.1371/journal.pone.0150630 (PMC4782991; doi:10.1371/journal.pone.0150630)
Supplement: S3 Table — (PDF) [file pone.0150630.s004.pdf]

**Table 3. Features Used in Emotion Prediction.**

| Feature Type | | | Description | Example |
|---|---|---|---|---|
| Entrainment | Association | | Association among users | $Etr(v_i \rightarrow v_j)$ |
| | Patterns | Dual | Reciprocal entrainment | $G(v_i, v_j)$* <br> $SG(v_i, v_j)$** |
| | | Single | Single entrainment | $G(v_i, v_j)$* <br> $f(\mathbf{D}_i)$** |
| Modality | Emotion | Uni-Gram | Emotion status at each timestamp | POS_1, NEG_2 … |
| | | Bi-Gram | Emotion dependence existing in different timestamps | POS_1-NEG_2 … |
| | Activity | Uni-Gram | User activity at each timestamp | Low_1, Medium_2, High_3, … |
| | | Bi-Gram | Activity correlation across different timestamps | Low_1-High_3, … |

Note: All features are extracted within a sliding window of 4 timestamps.
Numbers in features are relative positions to the timestamps for prediction.
* : features only used for EnFG; **: features only used for comparison methods.
